# Estimating cell diffusivity and cell proliferation rate by interpreting IncuCyte ZOOM™ assay data using the Fisher-Kolmogorov model

**DOI:** 10.1186/s12918-015-0182-y

**Published:** 2015-07-19

**Authors:** Stuart T. Johnston, Esha T. Shah, Lisa K. Chopin, D. L. Sean McElwain, Matthew J. Simpson

**Affiliations:** School of Mathematical Sciences, Queensland University of Technology (QUT), Brisbane, 4001 Australia; Ghrelin Research Group, Translational Research Institute, Institute of Health and Biomedical Innovation and APCRC-Q, QUT, 37 Kent St, Woolloongabba, 4102 Australia

**Keywords:** Cell motility, Cell proliferation, Scratch assay, Leading edge detection, Cancer, Wound healing

## Abstract

**Background:**

Standard methods for quantifying IncuCyte ZOOM™ assays involve measurements that quantify how rapidly the initially-vacant area becomes re-colonised with cells as a function of time. Unfortunately, these measurements give no insight into the details of the cellular-level mechanisms acting to close the initially-vacant area. We provide an alternative method enabling us to quantify the role of cell motility and cell proliferation separately. To achieve this we calibrate standard data available from IncuCyte ZOOM™ images to the solution of the Fisher-Kolmogorov model.

**Results:**

The Fisher-Kolmogorov model is a reaction-diffusion equation that has been used to describe collective cell spreading driven by cell migration, characterised by a cell diffusivity, *D*, and carrying capacity limited proliferation with proliferation rate, *λ*, and carrying capacity density, *K*. By analysing temporal changes in cell density in several subregions located well-behind the initial position of the leading edge we estimate *λ* and *K*. Given these estimates, we then apply automatic leading edge detection algorithms to the images produced by the IncuCyte ZOOM™ assay and match this data with a numerical solution of the Fisher-Kolmogorov equation to provide an estimate of *D*. We demonstrate this method by applying it to interpret a suite of IncuCyte ZOOM™ assays using PC-3 prostate cancer cells and obtain estimates of *D*, *λ* and *K*. Comparing estimates of *D*, *λ* and *K* for a control assay with estimates of *D*, *λ* and *K* for assays where epidermal growth factor (EGF) is applied in varying concentrations confirms that EGF enhances the rate of scratch closure and that this stimulation is driven by an increase in *D* and *λ*, whereas *K* is relatively unaffected by EGF.

**Conclusions:**

Our approach for estimating *D*, *λ* and *K* from an IncuCyte ZOOM™ assay provides more detail about cellular-level behaviour than standard methods for analysing these assays. In particular, our approach can be used to quantify the balance of cell migration and cell proliferation and, as we demonstrate, allow us to quantify how the addition of growth factors affects these processes individually.

## Background

Scratch assays are commonly used to quantify the potential for collective cell spreading by taking a spatially uniform population of cells on a two-dimensional substrate, creating an artificial scratch in the monolayer, and then making observations about the rate at which the remaining population spreads into the vacant region [1–10]. Scratch assays are routinely used since they are technically straightforward, fast and inexpensive [11]. Data obtained from scratch assays can be used to improve our understanding of drug design, malignant spreading and tissue repair [11].

A key limitation of scratch assays is the question of whether they are reproducible since the scratch can be made with various types of instruments and varying degrees of pressure, and the assay can be performed on several different types of substrates. All of these variables have the potential to affect the results of the scratch assay. Inspired by these limitations, new platforms to perform scratch assays, such as the IncuCyte™ and IncuCyte ZOOM™ real time live cell imaging assays have been developed [12]. IncuCyte ZOOM™ assays have the advantage that the scratch is reproducibly created with a mechanical tool and live images are obtained without the need to interrupt the experiment for imaging purposes [12].

**Fig. 1:**
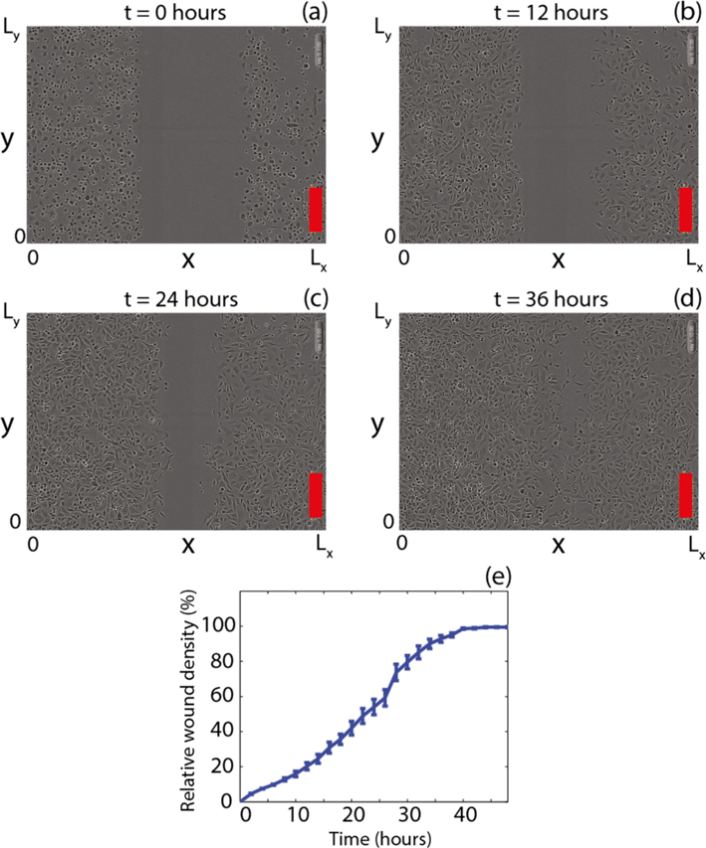
Images from the control IncuCyte ZOOM™ assay with PC-3 cells showing, (**a**) the initial position of the scratch, and the subsequent collective cell spreading after 12, 24 and 36 h in (**b**)–(**d**), respectively. Scale bar corresponds to 300 *μ*m. The results in (**e**) show the standard way of presenting IncuCyte ZOOM™ assay data for these experiments as the relative wound density as a function of time. Here we present the average relative wound density from *n*=3 identically prepared experimental replicates. The error bars in (**e**) indicate one standard deviation about the mean

Typical approaches to quantify IncuCyte ZOOM™ assay data involve making use of automated features that allow the user to quantify the proportion of the initially-scratched area that becomes re-colonised by cells as a function of time. As the assay proceeds and the cell population spreads into the initially-vacant area, the proportion of the area covered by cells increases with time. Typically, this data is presented as a plot of *relative wound density* as a function of time [13–15]. The relative wound density is a ratio of the occupied area of the initially-scratched area to the total area of the scratch [12]. To illustrate this typical approach we present a series of images from an IncuCyte ZOOM™ assay with PC-3 cells [16] in Fig. 1. PC-3 cells are a prostate cancer cell line with high metastatic potential [16, 17]. The experimental image in Fig. 1(a) shows the initial scratch, and the subsequent re-colonisation of the initially-vacant area is shown in Fig. 1(b)–(d). The data in Fig. 1(e) demonstrates the temporal variation in the relative wound density, which is automatically calculated by the IncuCyte ZOOM™ system [12]. While this kind of standard approach for quantifying IncuCyte ZOOM™ assays can provide useful information about how quickly a particular cell population is able to re-colonise the initially-vacant area, it does not distinguish between the relative roles of various cellular functions. The collective spreading of a population of cells is driven by both cell motility and cell proliferation [1–4, 18]. However, traditional data extracted from IncuCyte ZOOM™ assays does not give us any indication of the relative roles of cell motility and cell proliferation. This additional information could be important in terms of understanding how a particular growth factor or a potential drug treatment affects collective spreading since it is possible that the addition of a growth factor or drug treatment could affect: (i) cell motility alone, (ii) cell proliferation alone, or (iii) both cell motility and cell proliferation, simultaneously.

In this methodology article we describe an alternative method for interpreting IncuCyte ZOOM™ assay data using a continuum mathematical model. Our approach allows us to quantify the rate of cell migration in terms of an undirected cell diffusivity, *D*, and the rate of cell proliferation in terms of the proliferation rate, *λ*, and carrying capacity density, *K*. Applying this approach to a suite of IncuCyte ZOOM™ assay data using PC-3 prostate cancer cells allows us to obtain estimates of *D*, *K* and *λ* for these cells. Under control conditions our method gives *D*≈1.32×10^2^*μm*^2^/h, *K*≈1.13×10^−3^ cells/*μ*m^2^ and *λ*≈5.07×10^−2^ /h, which corresponds to a cell doubling time of approximately 14 h. We provide additional datasets where all experiments are repeated with varying concentrations of human epidermal growth factor (EGF) [19, 20], which leads to enhanced collective spreading. Applying our technique to this additional data indicates that the EGF acts affects both *D* and *λ*, but not *K*. In particular, our results suggest that *D* increases monotonically with EGF concentration whereas we observe a nonmonotonic relationship between *λ* and EGF concentration, with a maximum proliferation rate when the assays are treated with 50 ng/mL EGF. Although the techniques described here have been used previously to calibrate mathematical models to experimental data from circular barrier assays [18, 21, 22], this is the first time that IncuCyte ZOOM™ assay data has been used to calibrate these parameters, and the first time that this process has been used to quantify how estimates of *D*, *λ* and *K* depend on the concentration of EGF in an IncuCyte ZOOM™ assay.

## Methods

### IncuCyte ZOOM™ Assay

We perform a monolayer scratch assay using the IncuCyte ZOOM™ live cell imaging system (Essen BioScience, MI USA). This system measures scratch closure in real time and automatically calculates the relative wound density within the initially-vacant area at each time point. The relative wound density is the ratio of the occupied area to the total area of the initial scratched region. All experiments are performed using the PC-3 prostate cancer cell line [16], which is obtained from the American Type Culture Collection (ATCC, Manassas, USA). Cells are routinely propagated in RPMI 1640 medium (Life Technologies, Australia) in 10 % foetal calf serum (Sigma-Aldrich, Australia), with 110 u/mL penicillin, 100 *μ*g/mL streptomycin (Life Technologies), in plastic flasks (Corning Life Sciences, Asia Pacific) in 5 % CO _2_ and air in a Panasonic incubator (VWR International) at 37 °C. Cells are regularly screened for *Mycoplasma* (ATCC). Cells are removed from the monolayer using TrypLE™(Life Technologies) in phosphate buffered saline, resuspended in medium and seeded at a density of 20,000 cells per well in 96-well ImageLock plates (Essen BioScience). After seeding, cells are grown overnight to form a spatially uniform monolayer. We use a WoundMaker™(Essen BioScience) to create uniform, reproducible scratches in all the wells of a 96-well plate. After creating the scratch, the medium is aspirated and the wells are washed twice with fresh medium to remove any cells from the scratched area. Following the washes, for the control assay, 100 *μ*L of fresh medium is added to each well. We also perform a series of experiments where, following the washes, fresh medium containing different concentrations of EGF (Life Technologies) is added to the wells. The concentrations of EGF we use are: 25, 50, 75, 100 and 125 ng/mL. We will refer to these assays as EGF-25, EGF-50, EGF-75, EGF-100 and EGF-125, respectively. Once the fresh medium is added, the plate is placed into the IncuCyte ZOOM™ apparatus and images of the collective cell spreading are recorded every 2 hours for a total duration of 46 hours. For the control assay and each different EGF concentration we perform three identically prepared experimental replicates (*n*=3).

### Image analysis

We use Matlab’s Image Processing Toolbox [23, 24] to estimate the position of the leading edge of the spreading cell population in the IncuCyte ZOOM™ images. The experimental image is imported and converted to greyscale using the imread and rgb2gray commands, respectively. We detect edges in the images using edge with the Canny method [25] and automatically-selected threshold values. Detected edges outside of these threshold values are ignored. Remaining edges are dilated using the imdilate command and a structuring element, defined using strel, with a circular element of size 15. Using the bwareaopen command with a component size of 10,000 pixels, we remove any remaining vacant spaces in the image while preserving the vacant scratch. Edge dilation is reversed using the imerode command with the same structuring element defined previously to erode the image. Finally, edges within the image are smoothed using medfilt2 and the area of the remaining vacant space, *A*(*t*), representing the vacant area, is estimated using the regionprops command.

We calculate the position of the leading edge, which we define to be the distance between the centre of the experimental domain and the position of the leading edge using

(1) $$ L_{E}(t) = \frac{L_{x} L_{y} - A(t)}{2L_{y}},  $$

where *L*_*x*_ is the horizontal width of the image and *L*_*y*_ is the vertical height of the image. For all experiments we have *L*_*x*_=1970*μ*m and *L*_*y*_=1430*μ*m. Eq. (1) allows us to examine the time evolution of the scratched area in terms of *L*_*E*_(*t*), which is the half-width of the scratch (Fig. 1(a)).

### Mathematical model

We interpret our experimental results using the Fisher-Kolmogorov equation [26–28], which is a continuum reaction-diffusion model describing the spatiotemporal evolution of cell density in a population of cells where cell migration is driven by random (undirected) cell motility and cell proliferation is driven by carrying capacity limited logistic growth. The Fisher-Kolmogorov equation, and extensions of the Fisher-Kolmogorov equation, have been previously applied to *in vitro* [29–31] and *in vivo* [32, 33] data describing collective cell spreading in a range of contexts including wound healing [34, 35], tissue repair [3, 4] and malignant spreading [8–10, 36, 37].

Since our scratch assay takes place on a two-dimensional substrate (Fig. 1(a)–(c)), we start with the two-dimensional analogue of the Fisher-Kolmogorov equation in Cartesian coordinates

(2) $$ \begin{aligned} {}\frac{\partial \overline{C}(x,y,t)}{\partial t} = D \left(\frac{\partial^{2} \overline{C}(x,y,t)}{\partial x^{2}} + \frac{\partial^{2} \overline{C}(x,y,t)}{\partial y^{2}} \right) \\+ \lambda \overline{C}(x,y,t) \left(1 - \frac{\overline{C}(x,y,t)}{K} \right), \end{aligned}  $$

where $\overline {C}(x,y,t)$ [cells/ *μm*^2^] is the cell density, or average number of cells per unit area, at location (*x*,*y*) and time *t*. For our experiments we have 0≤*x*≤*L*_*x*_ and 0≤*y*≤*L*_*y*_. There are three parameters in the Fisher-Kolmogorov equation: (i) the cell diffusivity, *D* [ *μm*^2^/h], (ii) the cell proliferation rate, *λ* [/h], and (iii) the carrying capacity density, *K* [cells/ *μm*^2^]. The proliferation rate, *λ*, is related to the cell doubling time *t*_*d*_=log_e_(2)/*λ*. We note that we make the standard assumption that *D*, *λ* and *K* are constants [1–4, 29, 34]

Since the initial cell monolayer is spatially uniform and the initial scratch is made perpendicular to the *x*-direction (Fig. 1(a)), we can simplify the mathematical model by averaging in the *y*-direction [8–10]. To do this we average the two-dimensional cell density

(3) $$ C(x,t) = \frac{1}{L_{y}}\int_{0}^{L_{y}} \overline{C}(x,y,t) \; \mathrm{d}y,  $$

which allows us to write Eq. (2) as a one-dimensional partial differential equation

(4) $$ \frac{\partial C(x,t)}{\partial t} = D \frac{\partial^{2} C(x,t)}{\partial x^{2}} + \lambda C(x,t) \left(1 - \frac{C(x,t)}{K} \right).  $$

In general, approximating a two-dimensional nonlinear partial differential equation, such as Eq. (2), by an averaged one-dimensional nonlinear partial differential equation, such as Eq. (4), can introduce an averaging error. However, for initial conditions such as ours where the initial density is independent of the vertical direction, this error vanishes, and a detailed analysis of this error is presented elsewhere [38, 39]. The initial condition for Eq. (4) is given by the width of the scratch (Fig. 1(a))

(5) $$ {}C(x,0)= \left\{ \begin{array}{cp{1mm}l} C_{0} && {0 \le x < 985-L_{E}(0)\mu m}\,, \\ 0 && {985-L_{E}(0) \le x < 985+L_{E}(0)\mu m}\,, \\ C_{0} && {985+L_{E}(0) \le x \le 1970\mu m}\\ \end{array} \right.  $$

where *C*_0_ is the initial density of cells in the monolayer and *L*_*E*_(0) is the initial position of the leading edge. Since we use a WoundMaker™tool to create uniform scratches in all experimental replicates, the initial condition, given by Eq. (5), applies to all experiments and cannot be varied.

The physical distribution of cells in each experiment extends well-beyond the *L*_*x*_×*L*_*y*_ rectangular region imaged by the IncuCyte ZOOM™ apparatus. Therefore, since the cells are spatially uniform except for the scratched region, there will be no net flux of cells across the vertical boundaries along the lines *x*=0 and *x*=*L*_*x*_. We model this by using zero-flux boundary conditions

(6) $$\begin{array}{*{20}l} \frac{\partial C(x,t)}{\partial x} &= 0 \quad \text{at} \quad x=0, \notag \\ \frac{\partial C(x,t)}{\partial x} &= 0 \quad \text{at} \quad x=L_{x}. \end{array} $$

These boundary conditions do not imply that cells are stationary at *x*=0 and *x*=*L*_*x*_. Instead, these boundary conditions imply that the cell density profile is spatially uniform, *∂C*(*x*,*t*)/*∂x*=0, so that there is no net flux of cells across the boundaries at *x*=0 and *x*=*L*_*x*_.

We solve Eq. (4) using a finite difference numerical method [40]. The spatial domain, 0≤*x*≤*L*_*x*_, is discretised uniformly with grid spacing *δx*, and the spatial derivatives are approximated using a central-difference approximation [40]. This leads to a system of coupled nonlinear ordinary differential equations that are integrated through time using a backward-Euler approximation with constant time steps of duration *δt* [40]. The resulting system of coupled nonlinear algebraic equations are linearised using Picard (fixed-point) iteration, with absolute convergence tolerance *ε* [41]. The associated tridiagonal system of linear equations is solved using the Thomas algorithm [40]. For all results presented here we always chose *δx*, *δt* and *ε* so that our numerical algorithm produces grid-independent results.

We also apply Eq. (4) to some simplified situations where we focus on the time evolution of the cell density in small subregions, located well-behind the initial position of the scratch, where the cell density is approximately spatially uniform. This implies that *C*(*x*,*t*)≈*C*(*t*) within these subregions [18, 21, 22]. Since the cell density is approximately spatially uniform we have *∂C*(*x*,*t*)/*∂x*=0, and the first term on the right of Eq. (4) vanishes and, subsequently in these subregions, the partial differential equation simplifies to the logistic equation,

(7) $$ \frac{\text{d} C(t)}{\text{d} t} = \lambda C(t) \left(1 - \frac{C(t)}{K} \right),  $$

whose solution is given by

(8) $$ C(t) = \frac{K C(0)}{C(0) - \mathrm{e}^{-\lambda t} \left(C(0)-K \right)},  $$

where *C*(0)=*C*_0_ is the initial density at *t*=0. The simplification of approximating Eq. (4) by Eq. (7) in subregions well-behind the leading edge where the cell density is spatially uniform does not imply that cells are stationary in these subregions. Instead, Eq. (7) represents the situation where there is no gradient in cell density and cells are free to move amongst the extracellular space within these subregions. The key advantage of applying this approximation is that cell motion in these spatially uniform subregions does not contribute to any temporal changes in cell density. Instead, when the cell density is spatially uniform, any temporal change in cell density is solely associated with the proliferation term in Eq. (4) [18, 21, 22].

### Parameter estimation

We estimate the three parameters in the Fisher-Kolmogorov model using a sequential approach. First, using cell counting, we estimate the parameters governing cell proliferation: *K* and *λ*. Second, using data describing the temporal changes in the position of the leading edge, we estimate the cell diffusivity, *D*. Although it is possible to use a different approach, based on a multivariate regression technique to estimate *D*, *λ* and *K* simultaneously, we prefer to estimate these parameters sequentially. Estimating the three parameters sequentially, one at a time, emphasises the differences in the interpretation of these parameters, as well as emphasising the differences in the mechanisms of cell proliferation and cell motility. If, instead, a multivariate approach is used to estimate the three parameters simultaneously, we anticipate that the interpretation of the mechanisms associated with these parameters might not be obvious as it is in our approach.

#### Carrying capacity density

**Fig. 2:**
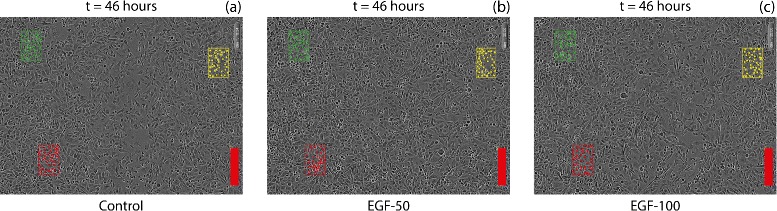
Final time experimental images (*t*=46 hours) for three IncuCyte ZOOM™ assays for (**a**) Control, (**b**) EGF-50, and (**c**) EGF-100. The three coloured boxes indicate the location of the three subregions used to estimate *K* and *λ*. Each coloured square within the subregions indicates the centre of an individual cell in the cell counting step. Scale bar corresponds to 300 *μ*m

To estimate *K* we focus on experimental images from the latter part of the experiment, *t*=46 h, where the cell population has grown to confluence. We identify three smaller subregions, located well-behind the initial leading edge, and count the number of cells within each subregion, *N*. To quantify the variability in our estimate we analyse three different subregions in each image and count *N* in each replicate subregion. Using this data we estimate the average carrying capacity density as

(9) $$ K = \frac{\langle N \rangle}{A_{SR}}  $$

where 〈*N*〉 is the average number of cells within the subregion of area *A*_*SR*_=3.789×10^4^*μm*^2^. To examine whether EGF has any impact on the carrying capacity density we estimate *K* for the control assay and for each experiment treated with a different EGF concentration. Figure 2 shows IncuCyte ZOOM™ images at *t*=46 h with the location of three subregions superimposed. The images in Fig. 2(a)–(c) show the control, EGF-50 and EGF-100 assays, respectively. We note that the location of all three subregions in each image is located well-behind the initial position of the scratch (Fig. 1(a)) so that after *t*=46 h the local density of cells within each subregion has grown to confluence. To quantify the variability in our estimate of *K*, we calculate the sample standard deviation for each EGF concentration, and report results as a mean value for *K*, with the variation in our estimate given by plus or minus one standard deviation about the mean. Results are summarised in Table 1.

**Table 1:** Estimated *K*, *λ*, *D* and *C*_0_ values for PC-3 cells for different EGF concentrations. Results are reported as a mean, with the estimate of the variability given in the parenthesis

Experiment	*K* (cells/ *μ* *m* ^2^)	*λ* (/h)	D (*μ* *m* ^2^/h)	*C*_0_ (cells/ *μ* *m* ^2^)
Control	1.13 × 10^−3^ (1.11 × 10^−3^−1.14 × 10^−3^)	5.07 × 10^−2^ (4.12 × 10^−2^−6.03 × 10^−2^)	1.32 × 10^2^ (1.05 × 10^2^−1.98 × 10^2^)	6.84 × 10^−4^ (5.77 × 10^−4^−7.91 × 10^−4^)
EGF-25	1.04 × 10^−3^ (1.01 × 10^−3^−1.07 × 10^−3^)	5.59 × 10^−2^ (4.40 × 10^−2^−6.79 × 10^−2^)	1.59 × 10^2^ (1.27 × 10^2^−2.38 × 10^2^)	6.42 × 10^−4^ (5.01 × 10^−4^−7.83 × 10^−4^)
EGF-50	1.12 × 10^−3^ (1.11 × 10^−3^−1.13 × 10^−3^)	6.94 × 10^−2^ (5.60 × 10^−2^−8.27 × 10^−2^)	1.53 × 10^2^ (1.21 × 10^2^−2.18 × 10^2^)	7.79 × 10^−4^ (6.32 × 10^−4^−9.26 × 10^−4^)
EGF-75	1.12 × 10^−3^ (1.11 × 10^−3^−1.13 × 10^−3^)	5.74 × 10^−2^ (5.44 × 10^−2^−6.05 × 10^−2^)	1.64 × 10^2^ (1.30 × 10^2^−2.44 × 10^2^)	6.81 × 10^−4^ (5.75 × 10^−4^−7.87 × 10^−4^)
EGF-100	1.16 × 10^−3^ (1.11 × 10^−3^−1.20 × 10^−3^)	6.13 × 10^−2^ (5.17 × 10^−2^−7.08 × 10^−2^)	2.06 × 10^2^ (1.65 × 10^2^−3.12 × 10^2^)	7.12 × 10^−4^ (6.10 × 10^−4^−8.15 × 10^−4^)
EGF-125	1.11 × 10^−3^ (1.09 × 10^−3^−1.12 × 10^−3^)	5.48 × 10^−2^ (5.35 × 10^−2^−5.62 × 10^−2^)	2.40 × 10^2^ (1.90 × 10^2^−3.51 × 10^2^)	7.68 × 10^−4^ (6.55 × 10^−4^−7.68 × 10^−4^)

#### Proliferation rate

**Fig. 3:**
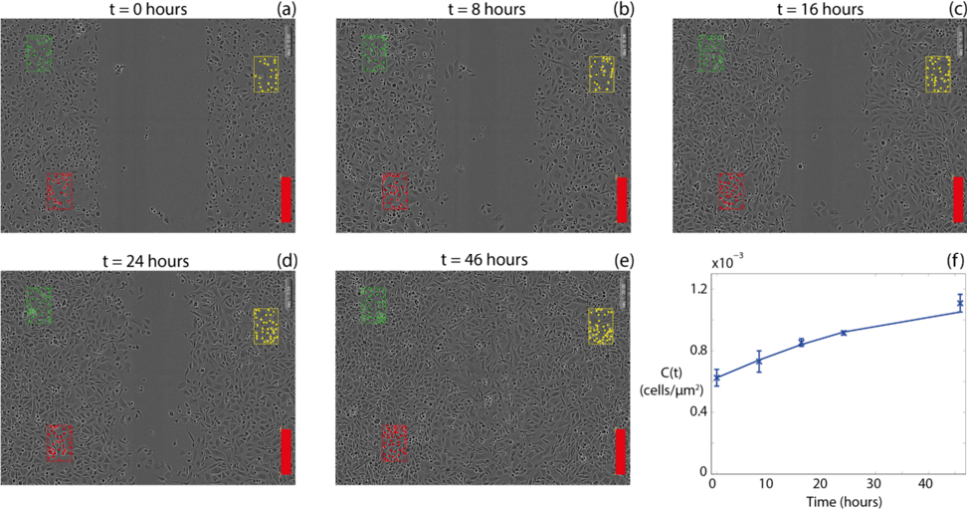
**a**-**e** Time evolution of an EGF-75 IncuCyte ZOOM™ assay. Images taken after (**a**) 0, (**b**) 8, (**c**) 16, (**d**) 24, and (**e**) 46 h after the scratch was performed. The three coloured boxes indicate the location of the three subregions used to calculate *K* and *λ*. Each coloured square within the subregions indicates the centre of an individual cell in the cell counting step. Scale bar corresponds to 300 *μ*m. **f** Comparison of the average experimental cell density *C*(*t*) (*crosses*) and the logistic growth curve using our estimates of *K* and *λ* (*solid*)

The logistic equation, given by Eq. (7), describes the time evolution of the cell density where there is, on average, no spatial variation in cell density. To apply the logistic equation to our data we analyse three subregions within each IncuCyte ZOOM™ image at several time points during the assay. Counting the total number of cells in each subregion and dividing by the area of the subregion gives an estimate of the local cell density in that subregion. In all cases the subregions we considered always started off with approximately 20–30 cells at *t*=0 h. Repeating this procedure for three different subregions, at fixed locations, for each experimental replicate, at five different time points, allows us to calculate the average cell density as a function of time, *C*(*t*). With this data, together with our previous estimates of *K*, we find the value of *λ* in Eq. (8) that matches our *C*(*t*) data across several time points. For consistency, when we estimate *λ* we always analyse the same three subregions that we used previously to estimate *K*. The location of these three subregions is shown in Fig. 3. We estimate the initial cell density, *C*(0), from the first image taken immediately after the scratch is made at *t*=0 h. Images of the assay in Fig. 3(a)–(e) correspond to 0, 8, 16, 24 and 46 h, respectively. The location of each subregion is chosen to be well-behind the initial position of the leading edge of the population so that the cell density is approximately spatially uniform locally within each subregion. In each subregion *C*(*t*) increases with time, and we attribute this increase to cell proliferation. The data in Fig. 3(f) shows the time evolution of the average cell density, *C*(*t*), calculated by averaging the three estimates of cell density from each subregion, at each time point. Using our previous estimate of *K*, we estimate *λ* by matching the solution of Eq. (7) with the observed *C*(*t*) data.

For each EGF concentration we have three sets of data describing the temporal variation in average cell density per experimental replicate. For each set of time series data we use Matlab’s lsqnonlin function, a nonlinear least squares minimisation routine [42], to estimate *λ*. To quantify the average proliferation rate we calculate the average *λ* value by averaging the three estimates from each experimental replicate. A comparison of the resulting logistic growth curve using our average estimate of *K* and *λ* with the observed *C*(*t*) data is given in Fig. 3(f) for the EGF-25 experiment, indicating that the solution of Eq. (7) matches the data reasonably well. To quantify the variability in *λ* we report our mean estimate of *λ* and the variation as the mean plus or minus one standard deviation. We also report the mean and variability in the *C*_0_ values used to obtain estimates of *λ*. Results are summarised in Table 1.

#### Cell diffusivity

Several different approaches have been used in previous studies to estimate *D* from *in vitro* assays describing collective cell spreading processes. For example, Treloar et al. [22] estimate *D* in a circular barrier assay by applying two different methods to the same data set. First, they estimate *D* using a cell labelling and cell counting technique to provide an estimate of the cell density profiles near the leading edge of the spreading population. Treloar et al. [22] calibrate the solution of a partial differential equation to that data to give an estimate of *D* which matches the position and shape of the spreading cell density profile. Second, using the same data set, Treloar et al. [22] use automated leading edge image analysis [23, 24] to quantify temporal changes in the position of the leading edge of the spreading cell density profile without counting individual cells. Treloar et al. [22] calibrate their model to this leading edge data to obtain a second estimate of *D*. Given that the two approaches implemented by Treloar et al. [22] produce similar estimates of *D*, here we choose to estimate *D* using a similar technique based on leading edge data since this is the most straightforward approach which avoids the need for labelling and counting individual cells within the spreading population.

**Fig. 4:**
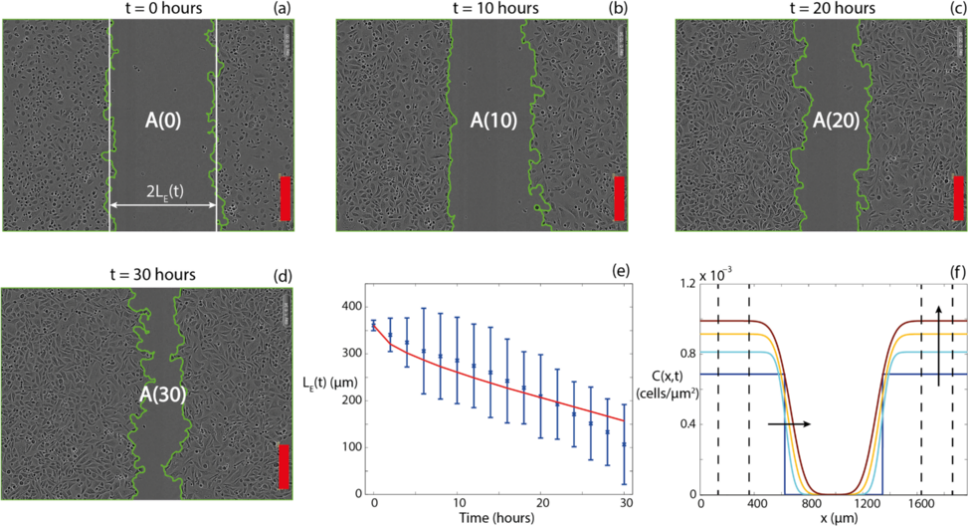
**a**-**d** Indicate the area of remaining vacant space, *A*(*t*), as determined by the edge detection algorithm at **a** 0, **b** 10, **c** 20, and **d** 30 h for the control assay. The position of the detected leading edge is given in green. The straight vertical lines superimposed on **a** (*white*) indicate the average width of the scratch, 2*L*_*E*_(*t*). Scale bar corresponds to 300 *μ*m. **e** Average *L*_*E*_(*t*) data estimated from the control assay experimental images (*blue*). The error bars correspond to one standard deviation about the mean. Numerical *L*_*E*_(*t*) data (*red*), corresponding to the numerical solution of Eq. (4) using the relevant estimates of *D*, *λ* and *K* (Table 1). (**f**) Evolution of *C*(*x*,*t*) profiles at *t*=0, 10, 20, 30 h corresponding to the numerical solution of Eq. (4) using the relevant estimates of *D*, *λ* and *K* (Table 1). Arrows indicate the direction of increasing time. Numerical solutions of Eq. (4) correspond to *δ* *x*=1*μ*m, *δ* *t*=0.1 h and *ε*=1×10^−6^. The vertical lines show the locations of the subregions where the estimates of *λ* and *K* were obtained

Figures 4(a)-(d) show IncuCyte ZOOM™ images at 0, 10, 20 and 30 h, respectively. The position of the two detected leading edges of the spreading population is superimposed on each image. A visual comparison of how the position of the detected leading edges changes with time suggests that the initially-vacant region closes symmetrically with time. The edge detection results allow us to calculate the area of the vacant region, *A*(*t*), and with this information Eq. (1) allows us to estimate the half-width, *L*_*E*_(*t*), which is decreasing function of time. Previously, Treloar et al. [22, 24] showed that the location of the automatically detected leading edge corresponds to a cell density of approximately 2 % of the carrying capacity density.

Given our previous estimates of *K* and *λ*, and assuming that the position of the detected leading edge corresponds to the location where the density is 2 % of the carrying capacity, we use Matlab’s lsqnonlin function to find an estimate of *D* that minimises the difference between the observed time series of *L*_*E*_(*t*) and the time series of *L*_*E*_(*t*) data from the numerical solution of Eq. (4). We present an example of the match between the experimental measurements of *L*_*E*_(*t*) and numerical prediction of *L*_*E*_(*t*) in Fig. 4(c). For all time points, the numerical estimate of *L*_*E*_(*t*) is always within one standard deviation of the mean of the experimental measurements. Given our estimates of *D*, *λ* and *K*, we can use our numerical solution of Eq. (4) to explore how *C*(*x*,*t*) varies across the entire width of the domain, for the duration of the assay, as illustrated in Fig. 4(f). These profiles show that the cell density remains approximately spatially uniform well-behind the initial location of the scratch. In fact, we have indicated the position of the location of the various subregions used to estimate *K* and *λ* on the profiles in Fig. 4(f), and we see that the predicted cell density profile is spatially uniform, *∂C*(*x*,*t*)/*∂x*=0, at these locations for the duration of the assay, which is visually consistent with the subregions presented in Fig. 2. The cell density in the three subregions well-behind the initial location of the scratch increases with time owing to cell proliferation. These profiles also show the cell density front near the location of the scratch moves inward to close the initially-scratched region with time.

Using our approach we calculate an average value of *D* by estimating the cell diffusivity for each experimental replicate and then averaging the results. We note that Treloar et al. found that varying the Matlab edge detection threshold parameters led to a small variation in the position of the detected leading edge corresponding to a cell density in the range of approximately 1–5 % of the carrying capacity density [24]. To quantify the variability in our estimate of *D* we repeated the edge detection by assuming that the position of the detected leading edge corresponds to both 1 and 5 % of the carrying capacity density. Results are summarised in Table 1. We note that the maximum value of *D* is obtained by assuming that the detected leading edge corresponds to 5 % of the carrying capacity since this upper bound implies additional spreading. Conversely, the minimum value of *D* is obtained by assuming that the detected leading edge corresponds to 1 % of the carrying capacity since this lower bound implies less spreading.

## Results

By applying the parameter estimation procedures described previously, we obtain estimates of *K*, *λ* and *D*, as well an estimates of the variability in these values. These results are summarised in Table 1. Comparing our estimates of *K*, *λ* and *D* for each assay with a different concentration of EGF provides us with information about how EGF affects cell proliferation and cell motility for the PC-3 cell line. Data in Table 1 indicates that *K* varies by no more than approximately 8 % between the different experiments with different EGF concentrations relative to the control experiment. In comparison, our estimates of *λ* and *D* vary by approximately 37 % and 82 %, respectively, between different experiments with different EGF concentrations, relative to the control experiment. These results imply that EGF affects both the rate of cell motility and the rate of cell proliferation; however, EGF appears to have a smaller influence on the carrying capacity density, suggesting that it has minimal impact on the physical shape and maximum packing density of PC-3 cells.

Results in Table 1 suggest that we observe an increase in the rate of proliferation with small concentrations of EGF. However, there appears to be a reduction in the rate of cell proliferation at larger concentration of EGF, implying that there is an optimal stimulation of proliferation at an EGF concentration of 50 ng/mL. This kind of nonmonotonic response to EGF has been observed in previous experiments involving both PC-3 [17] and other cell types [43]. However, unlike these previous studies [17, 43], our approach allows us to estimate how EGF affects both cell migration and cell proliferation separately.

Results in Table 1 suggest that EGF significantly enhances cell motility, *D*. However, unlike the response in *λ*, our results suggest that the diffusivity of PC-3 cells appears to be an monotonically increasing function of EGF for the concentrations of EGF that we consider.

## Discussion and conclusion

In this work we provide an alternative method for analysing IncuCyte ZOOM™ assays [12]. The traditional approach for analysing IncuCyte ZOOM™ assays is to report the temporal variation in the relative wound density (Fig. 1), which is the ratio of the occupied area to the initially-vacant area of the scratch [13–15]. While this data allows us to quantify the rate of collective cell spreading, it does not provide any quantitative insight into the relative roles of different mechanisms that drive collective cell spreading. As an alternative, we present a method which allows us to analyse standard images from IncuCyte ZOOM™ assays by interpreting the results quantitatively using the Fisher-Kolmogorov equation [26, 27]. Our approach provides a quantitative measure of the relative roles of cell migration and cell proliferation by estimating the carrying capacity density, *K*, the cell proliferation rate *λ*, and the cell diffusivity, *D*.

To estimate *K* we focus on images from the latter part of the IncuCyte ZOOM™ assay, *t*=46 h, by which time the cell monolayer has grown to confluence. We count the number of cells in several subregions located well-behind the initial position of the scratch. Dividing the cell counts by the area of the subregion gives us an estimate of the carrying capacity density, *K*. To examine the influence of our choice of the area of the subregion, we also examine the sensitivity of our estimate of *K* for the control assay to variations in the area of the subregion. For example, with *A*_*SR*_=3.789×10^4^*μm*^2^, we obtain *K*=1.13×10^−3^±0.01×10^−3^ cells/ *μm*^2^ for the control assay. Repeating the procedure and doubling *A*_*SR*_ gives an estimate of *K* which is within 2 % of the original estimate. Therefore, our estimate of *K* is practically insensitive to the size of the subregion.

To estimate *λ* we count the numbers of cells in several subregions, located well-behind the initial position of the scratch, at several time points during the assay. This allows us to quantify how the cell density behind the initial scratch increases with time to reach carrying capacity density. Using this information we calibrate the solution of the logistic equation, using our previous estimate of *K*, to that data, which provides an estimate of *λ*. To estimate *λ* we focus on relatively early time data, *t*=0,8,16,24 and 46 h, since we anticipate that most of the proliferation activity occurs before the cell population reaches confluence. Using this approach for the control assay we obtain *λ*=5.07×10^−2^±0.96×10^−2^ /h. To examine the influence of our choice of time points we re-estimate *λ* using data at *t*=0,8,16,24,30 and 46 h, giving *λ*=5.53×10^−2^ /h, which is well-within the variability of the original estimate. Since the process of identifying and counting cells in various subregions is the most time-consuming aspect of our method together with the fact that including additional data at these intermediate times does not significantly alter our estimates of *λ*, we conclude that our choice of focusing on relatively early-time observations is adequate to provide estimates of *λ*.

Given our estimates of *λ* and *K*, we then estimate *D* by solving the Fisher-Kolmogorov equation numerically and finding a value of *D* which provides the best match between the position of the leading edge observed in the experiments and the position of the leading edge predicted by the numerical solution of the Fisher-Kolmogorov equation. Using this approach, for our control assays, we estimate *D*≈1.32×10^2^*μm*^2^/h, *λ*≈5.07×10^−2^/h and *K*≈1.13×10^−3^ cells/ *μm*^2^ for the PC-3 prostate cancer cell line [16]. Since typical values of *D* reported in the literature vary in the range of approximately 10^1^ - 10^3^*μm*^2^/h [3, 4, 21, 22, 29], our estimate of *D* seems reasonable since it is well within previously reported values for different cell lines.

In addition to analysing the control assay, we also estimate *D*, *λ* and *K* for a suite of assays where the cell culture medium is supplemented with different concentrations of EGF (25, 50, 75, 100 and 125 ng/mL) [19, 20]. Using our approach we estimate *D*, *λ* and *K* for each EGF concentration, which provides insight into how EGF affects the rate of cell migration, the rate of cell proliferation and the carrying capacity density for PC-3 cells in these assays. In summary, we find there is no consistent trend in our estimates of *K* with the different EGF treatments. The maximum variability in our estimate of *K* between different EGF concentrations is approximately 8 %, indicating that the carrying capacity density is relatively unaffected by EGF. In contrast, we find that our estimates of *D* and *λ* are both sensitive to EGF. The maximum variability in *D* and *λ* amongst different EGF treatments is approximately 82 % and 37 %, respectively. Therefore, our analysis suggests that EGF affects both cell motility and cell proliferation, with the impact on cell motility being more pronounced than the impact on cell proliferation. Interestingly our results suggests that we have a monotonic increase of *D* with EGF concentration whereas we have a nonmonotonic relationship between *λ* and EGF concentration. We observe a maximum stimulation of proliferation at an EGF concentration of 50 ng/mL.

Similar to other applications of the Fisher-Kolmogorov equation [1–4, 21, 29, 31, 34], we have made the standard assumption that the parameters in each experiment, *D*, *λ* and *K*, are constants which do not vary with position, time or cell density. Recently, there has been considerable interest in the theoretical physics and applied mathematics literature regarding the analysis of extensions of the Fisher-Kolmogorov equation where *D* and *λ* vary with position, time or cell density [44, 45]. Although these extensions are mathematically interesting, we have not attempted to apply such an extension here since the precise form of the putative spatial or temporal dependence is unknown, and at this stage, we anticipate that more detailed experimental data would be required to calibrate these more detailed mathematical models. We leave this extension as a potential topic for future analysis.

The question of whether there is any role for chemotaxis in this particular IncuCyte ZOOM™ assay has not been addressed in this work. Since we have been to obtain reasonable estimates for *D*, *λ* and *K* by calibrating the solution of the Fisher-Kolmogorov equation to our experimental data it is not obvious that we need to consider applying a more complicated model incorporating chemotactic cell migration at this time. However, it is possible that the cells produce a chemical signal, *G*(*x*,*t*), which as a result of diffusion and decay, could lead to the formation of a chemical gradient that stimulates additional directed cell motion [46]. An extension of the Fisher-Kolmogorov model which incorporates these effects can be written as [10, 28, 46, 47]

(10) $$\begin{array}{*{20}l} \frac{\partial C(x,t)}{\partial t} &= D \frac{\partial^{2} C(x,t)}{\partial x^{2}} - \chi \frac{\partial}{\partial x} \left(C(x,t) \frac{\partial G(x,t)}{\partial x} \right) \notag \\ & + \lambda C(x,t) \left(1 - \frac{C(x,t)}{K} \right), \end{array} $$

(11) $$\begin{array}{*{20}l} \frac{\partial G(x,t)}{\partial t} &= D_{g} \frac{\partial^{2} G(x,t)}{\partial x^{2}} + k_{1} C(x,t) - k_{2} G(x,t), \end{array} $$

where *χ* is the chemotactic sensitivity coefficient, *D*_*g*_ is the diffusivity of the chemotactic chemical, *k*_1_ is the rate at which cells produce the chemotactic chemical, and *k*_2_ is the rate at which the chemotactic chemical undergoes natural decay. This model can be used to simulate chemoattraction by setting *χ*>0 or chemorepulsion by setting *χ*<0 [28, 46, 47]. Comparing this chemotactic extension of the Fisher-Kolmogorov equation with the standard model, Eq. (4), indicates that there are an additional four parameters to estimate in order to apply the chemotaxis model: *χ*, *D*_*g*_, *k*_1_ and *k*_2_. Given that standard applications of the IncuCyte ZOOM™ assay do not attempt to make any measurement of the presence of any putative chemotactic factor, *G*(*x*,*t*) [13–15], nor have we made any measurements of *χ*, *D*_*g*_, *k*_1_ or *k*_2_, we do not attempt to calibrate this more complicated chemotaxis model to our IncuCyte ZOOM™ assay data. Instead, we suggest that if this kind of chemotaxis model were to be applied to an IncuCyte ZOOM™ assay data set, additional experimental measurements of these kinds of details are warranted.

## Availability of supporting data

The data set supporting our results is included within the article and the supplementary material document.

## Abbreviations

EGF: Epidermal growth factor
